# Glycoprotein YKL-40 Levels in Plasma Are Associated with Fibrotic Changes on HRCT in Asbestos-Exposed Subjects

**DOI:** 10.1155/2017/1797512

**Published:** 2017-05-14

**Authors:** Tuija Väänänen, Lauri Lehtimäki, Katriina Vuolteenaho, Mari Hämäläinen, Panu Oksa, Tuula Vierikko, Ritva Järvenpää, Jukka Uitti, Hannu Kankaanranta, Eeva Moilanen

**Affiliations:** ^1^The Immunopharmacology Research Group, Faculty of Medicine and Life Sciences, University of Tampere and Tampere University Hospital, P.O. Box 100, 33014 Tampere, Finland; ^2^Allergy Centre, Tampere University Hospital and Faculty of Medicine and Life Sciences, University of Tampere, P.O. Box 100, 33014 Tampere, Finland; ^3^Clinic of Occupational Medicine, Tampere University Hospital, P.O. Box 486, 33101 Tampere, Finland; ^4^Finnish Institute of Occupational Health, P.O. Box 486, 33101 Tampere, Finland; ^5^Department of Radiology, Tampere University Hospital, P.O. Box 2000, 33521 Tampere, Finland; ^6^Occupational Health, Faculty of Medicine and Life Sciences, University of Tampere, P.O. Box 100, 33014 Tampere, Finland; ^7^Department of Respiratory Medicine, Seinäjoki Central Hospital, 60220 Seinäjoki, Finland

## Abstract

YKL-40 is a chitinase-like glycoprotein produced by alternatively activated macrophages that are associated with wound healing and fibrosis. Asbestosis is a chronic asbestos-induced lung disease, in which injury of epithelial cells and activation of alveolar macrophages lead to enhanced collagen production and fibrosis. We studied if YKL-40 is related to inflammation, fibrosis, and/or lung function in subjects exposed to asbestosis. Venous blood samples were collected from 85 men with moderate or heavy occupational asbestos exposure and from 28 healthy, age-matched controls. Levels of plasma YKL-40, CRP, IL-6, adipsin, and MMP-9 were measured with enzyme-linked immunosorbent assay (ELISA). Plasma YKL-40 levels were significantly higher in subjects with asbestosis (*n* = 19) than in those with no fibrotic findings in HRCT following asbestos exposure (*n* = 66) or in unexposed healthy controls. In asbestos-exposed subjects, plasma YKL-40 correlated negatively with lung function capacity parameters FVC (Pearson's *r* −0.259, *p* = 0.018) and FEV_1_ (Pearson's *r* −0.240, *p* = 0.028) and positively with CRP (Spearman's rho 0.371, *p* < 0.001), IL-6 (Spearman's rho 0.314, *p* = 0.003), adipsin (Spearman's rho 0.459, *p* < 0.001), and MMP-9 (Spearman's rho 0.243, *p* = 0.025). The present finding suggests YKL-40 as a biomarker associated with fibrosis and inflammation in asbestos-exposed subjects.

## 1. Introduction

Asbestosis is a chronic interstitial fibrosing lung disease developing slowly after exposure to asbestos fibers. As the clearance of these fibers is very slow, exposure to asbestos can lead to chronic inflammatory changes and eventually to clinically detectable fibrosis after a latent period. In the pathogenesis of asbestosis, fibrosis is located first at the site of asbestos bodies, where macrophages accumulate and inflammatory reaction takes place, followed by a more diffuse fibrosis in the lungs, which is characterized by apoptosis of epithelial cells, fibroblast proliferation, and collagen deposition [1].

Asbestosis is classified as a rare disease by Orphanet portal for rare diseases [2] (i.e., it is affecting less than one person per 2000 in the European population, as defined by the EU Commission Public Health Policy on Rare Diseases [3]). In western countries, the use of asbestos has been banned or restricted in the developed countries starting from 1990s: asbestos is not anymore used in construction work today, and the risks of asbestos fibers in renovation are acknowledged [4, 5]. As fibrosis manifests 15 to 40 years after exposure to asbestos and highest level of asbestos use took place in the 1970s and 1980s in European countries, peak in the incidence of asbestos-related diseases is now levelling off [1, 4]. In contrast, asbestos use is still significant or increasing in countries such as Brazil, China, India, Iran, Kazakhstan, Russia, Thailand, and Ukraine [4]. The World Health Organization (WHO) has estimated that about 125 million people are still exposed to asbestos at the workplace and half of the deaths from occupational cancer are caused by asbestos [5, 6]. Measures to prevent exposure to asbestos are the most efficient way to eliminate these diseases, but the WHO calls also for improvements in diagnostics and treatment. Investigation of inflammatory and fibrotic processes in asbestosis is needed to allow drug development and to find novel biomarkers to diagnose and follow up these diseases [6].

YKL-40, a chitin-binding glycoprotein without the catalytic activity characteristic to the true chitinases, is related to various inflammatory and tissue-remodeling diseases. YKL-40 and its homologues are known by various names such as chitinase-3-like protein 1 (Chi3-l1), breast regression protein 39 (BRP-39), human cartilage glycoprotein 39 (HC gp-39), and chondrex [7, 8]. YKL-40 has been shown to associate with fibrosis and macrophage activation [8–10]. Increased circulating levels of YKL-40 were reported in fibrotic liver disease: high YKL-40 levels are associated with histologically more severe fibrotic changes in chronic alcoholic hepatitis, primary biliary cirrhosis, autoimmune hepatitis-induced cirrhosis, and hepatitis C [11–14], and YKL-40 was described as a marker of treatment response in interferon-treated patients with hepatitis C [14]. High YKL-40 levels have also been shown to associate with various forms of interstitial lung diseases (ILD), such as idiopathic pulmonary fibrosis (IPF), idiopathic nonspecific interstitial pneumonia (iNSIP), and cryptogenic organizing pneumonitis (COP) [15–17]. The aim of the present study was to investigate the hypothesis that YKL-40 is related to inflammation, fibrosis, and/or lung function in asbestos-exposed subjects.

## 2. Materials and Methods

### 2.1. Subjects

The study subjects (one hundred and eighteen men) were recruited among individuals with known history of moderate or heavy occupational exposure to asbestos who were therefore followed up at the Clinic of Occupational Medicine at Tampere University Hospital [18]. All of them were nonsmokers or had quit over five years previously. Thirty-three men were excluded due to meeting the exclusion criteria that were asthma or asthma medication, FEV_1_/FVC < 0.7, and bronchiectasis or emphysema on high-resolution computed tomography (HRCT) of the chest. The remaining 85 men formed the asbestos-exposed group in the study, and they were further divided into two groups according to the HRCT findings: 66 had normal lung parenchyma (HRCT class 0) or only minor borderline fibrotic findings (HRCT class 1), and 19 had bilateral parenchymal fibrosis, that is, asbestosis (HRCT classes 2–5), see below. The control group was recruited from the community and consisted of 28 healthy nonsmoking men with no respiratory symptoms and normal lung function. This study was approved by the Ethics Committee of Tampere University Hospital. All the subjects gave their written informed consent.

### 2.2. HRCT

HRCT was scanned (Siemens Somatom Plus 4; Siemens Medical, Erlangen, Germany) with 1 mm slices taken at 3 cm intervals using imaging parameters of 130–140 kV and 100–111 mA. The HRCT images were scored using consensus reading by two experienced thoracic radiologists as described previously [19, 20]. Findings indicating interstitial lung fibrosis (septal thickening, subpleural lines, parenchymal bands, or honeycombing) in both lungs were semiquantitatively scored according to a scale of classes from 0 to 5. Class 0 represents normal parenchymal finding, class 1 represents borderline parenchymal finding with minor sporadic changes only, and classes 2 to 5 represent mild to extreme diffuse pulmonary fibrosis [19].

### 2.3. Measurements of Circulating Biomarkers

Venous blood samples were drawn, and plasma/serum samples were stored at −70°C until analyzed. Enzyme-linked immunosorbent assay (ELISA) was performed using commercial reagents for YKL-40, adipsin, MMP-9, CRP (R&D Systems Europe Ltd., Abingdon, U.K.), and IL-6 (Sanquin, Amsterdam, The Netherlands).

### 2.4. Statistical Analysis

Distribution of plasma YKL-40 was skewed (Kolmogorov-Smirnov's test), and log transformation was used in statistical calculations to guarantee normally distributed data when needed. Correlations were calculated using Pearson's *r* between normally distributed variables and Log-YKL-40 and by using Spearman's rho between nonnormally distributed variables and YKL-40. Normally distributed data are presented as mean (SD) and skewed data as median (interquartile range, IQR). Comparison between groups was performed with unpaired *t*-test, Mann–Whitney *U* test, or one-way ANOVA with least significant difference (LSD) posttest where appropriate. *p* values less than 0.05 were considered significant. SPSS Statistics 23 software (SPSS Inc., Chicago, IL, USA) was used in the statistical analysis.

## 3. Results

Subject characteristics, lung function parameters, and circulating concentrations of inflammatory and fibrosis markers are given in Table 1. Based on HRCT findings, 66 of the 85 asbestos-exposed subjects had normal lung parenchyma (HRTC class 0) or only minor borderline fibrotic changes (HRTC class 1) and 19 had bilateral parenchymal fibrosis, that is, asbestosis (HRCT classes 2–5) [20]. Asbestos-exposed subjects with asbestosis were older than exposed subjects without fibrotic changes (*p* = 0.015, Table 1). However, there was no difference in the time since the beginning of the exposure between these two groups (Table 1) and YKL-40 did not correlate with age (*r* = 0.102, *p* = 0.355, Table 2). In subjects with asbestosis, diffusing capacity for carbon monoxide (*D*_L,CO_) was decreased by 18% compared to asbestos-exposed subjects without HRCT findings, but there was no difference between these groups in FVC or FEV_1_. Adipsin and MMP-9 levels were increased in subjects with asbestosis (Table 1).

**Figure 1:**
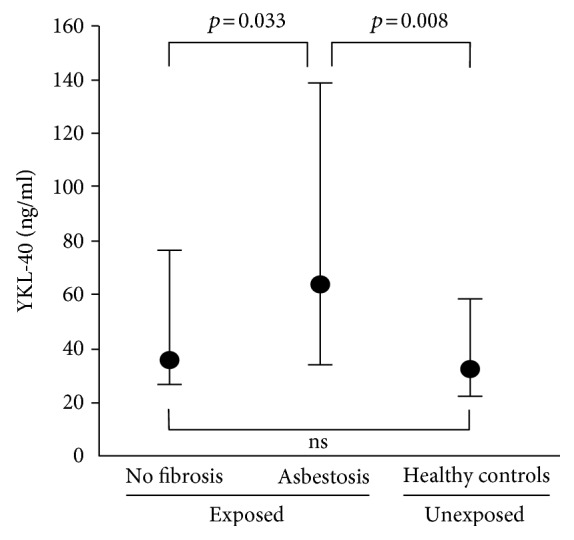
Plasma YKL-40 concentrations in subjects with asbestosis (*n* = 19), asbestos-exposed subjects who did not develop lung fibrosis (*n* = 66), and healthy controls (*n* = 28). Medians and interquartile ranges (IQR) of the three groups are shown. One-way ANOVA with least significant difference (LSD) posttest was used. ns: not significant.

Plasma YKL-40 concentration (median and IQR) was higher in subjects with asbestosis (64.4, 35.1–138.1 ng/ml) than in asbestos-exposed subjects without asbestosis (36.4, 27.3–76.7 ng/ml, *p* = 0.033) or in unexposed controls (32.8, 23.5–59.3 ng/ml, *p* = 0.008), as presented in Figure 1. In asbestos-exposed subjects, fibrotic changes detected on HRCT varied from 0 to 4, and in 32 of the 85 subjects, no changes were observed (i.e., were classified as 0). YKL-40 showed a positive correlation with the degree of developing/fibrotic changes (from 0.5 to 4, *n* = 53, rho = 0.392, *p* = 0.005).

**Figure 2:**
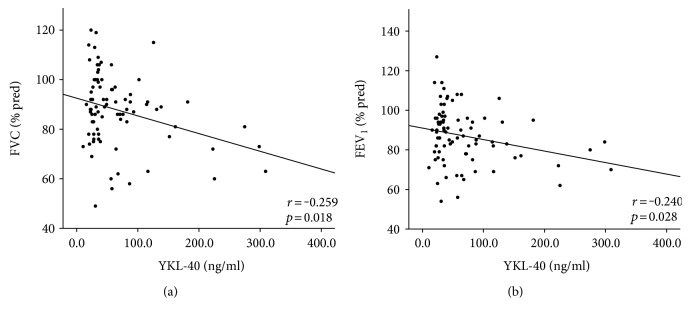
Correlations between YKL-40 and measures of respiratory function in asbestos-exposed subjects (*n* = 85). YKL-40 correlated with (a) FVC: forced vital capacity and (b) FEV_1_: forced expiratory volume in 1 second. Pearson's correlation coefficient was used because of skewed distribution of the data.

**Figure 3:**
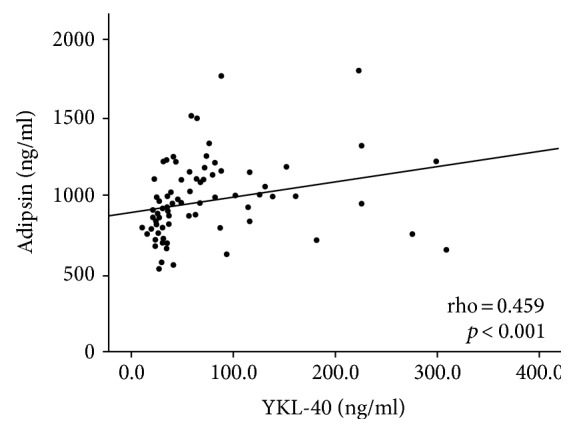
Correlation between YKL-40 and fibrosis marker adipsin in asbestos-exposed subjects (*n* = 85). YKL-40 correlated positively with adipsin. Spearman's correlation coefficient was used because of skewed distribution of the data.

To further investigate the association of YKL-40 with asbestosis, we assessed the correlations between YKL-40 and lung function indices, inflammatory, and fibrosis markers (Table 2). YKL-40 was found to correlate negatively with lung function capacity parameters FVC and FEV_1_ (Figure 2) and positively with inflammation markers CRP and IL-6, as well as with fibrosis markers adipsin and MMP-9, adipsin showing the strongest correlation (rho = 0.459, *p* < 0.001, Figure 3).

## 4. Discussion

To our knowledge, the present study is the first to show increased plasma YKL-40 levels in subjects with asbestosis. Circulating YKL-40 levels were significantly higher in subjects with asbestosis compared to subjects who did not develop lung fibrosis after moderate to heavy exposure to asbestos or to healthy controls. Moreover, in the subjects exposed to asbestos, plasma YKL-40 was negatively associated with lung function parameters (FVC and FVE_1_) and positively with biomarkers of inflammation and tissue injury suggesting a role for YKL-40 in the formation of pulmonary fibrosis following exposure to asbestos.

Supporting our findings, previous studies have shown increased levels of YKL-40 in other fibrotic pulmonary diseases. Increased levels of YKL-40 have been shown in patients with idiopathic pulmonary fibrosis, IPF [9, 15, 21], in which high YKL-40 levels were associated with progression of the disease [9, 21]. YKL-40 levels have been shown to associate also with other idiopathic interstitial lung diseases including nonspecific interstitial pneumonia, smoking-related interstitial lung disease, and cryptogenic organizing pneumonia [16], pulmonary sarcoidosis [22], posttransplantation bronchiolitis obliterans [23], and pulmonary manifestations of cystic fibrosis or systemic sclerosis [24–26]. Corradi et al. reported increased YKL-40 levels also in patients with malignant mesothelioma (*n* = 50), a disease often associated with asbestos exposure [27]. These studies support our results that high levels of YKL-40 are associated with the pathogenic process in asbestosis and other fibrotic pulmonary diseases.

The effects of asbestos exposure depend on several factors including the intensity and duration of the exposure, fiber type and size, and susceptibility of the exposed individual. Asbestosis is related especially to long (>20 *μ*m) fibers, and low-dose exposure associates with a macrophage-dominant immune response whereas high doses of asbestos lead to neutrophil-dominant inflammation. Ingestion of asbestos activates macrophages, in a pattern typical for alternatively activated M2 macrophages related to wound healing and fibrosis, to produce growth factors and cytokines that promote collagen formation in the fibroblasts [1, 28]. Intriguingly, YKL-40 is produced by human monocyte-derived differentiated macrophages [10] and has been suggested as a marker of alternatively activated M2 macrophages [28]. In peripheral blood of IPF patients, high levels of circulating YKL-40 were accompanied with M2-skewed gene expression profile in the peripheral blood mononuclear cells (PBMCs) [9]. Moreover, YKL-40 was a direct stimulator of alternative activation in mice alveolar and peritoneal macrophages [29]. In asbestos-exposed rats, alveolar macrophages were shown to have altered phenotype with long survival and high growth factor production resulting in fibrogenesis [30]. These findings suggest YKL-40 as a marker of M2 macrophage-driven fibrogenic processes in asbestosis, which could be potentially preventable by affecting YKL-40 levels.

In addition to its role in alternative activation of macrophages, there are several other possible effector functions for YKL-40 in asbestosis. Asbestos is known to induce formation of reactive oxygen species (ROS) in macrophages, on the surface of asbestos fibers and in the mitochondria of several cell types contributing to DNA damage and subsequent pulmonary toxicity through oxidative stress [1]. YKL-40, in turn, has been suggested to protect the lung by inhibiting oxidant-induced injury, vascular permeability, and apoptosis [8]. Moreover, YKL-40 is able to bind collagen types I, II, and III and to have modulatory effects on collagen fibrillation and collagenolytic cleavage, which could play a part in the fibrotic process [31].

In the present study, to assess the role of YKL-40 in asbestosis, we examined its associations with markers known to relate to the disease. YKL-40 showed positive correlations to markers of inflammation and fibrosis, supporting the view that YKL-40 is linked to the pathogenesis of asbestosis. First, plasma YKL-40 correlated with inflammation markers CRP and IL-6 in asbestos-exposed subjects. This is supported by previous studies showing similar findings in patients with heart transplantation, atrial fibrillation, type 2 diabetes, or rheumatoid arthritis [32–35] and in subjects during ongoing dialysis treatment [36]. Moreover, IL-6 has previously been shown to stimulate YKL-40 secretion from human chondrocytes [37] and human bone marrow-derived stem cells [38]. Circulating levels of IL-6 have been shown to be increased in asbestos-exposed subjects [39, 40] and suggested to be secreted from type II alveolar cells [39]. However, macrophages have been implicated also as a possible source for IL-6 [10, 41].

Secondly, YKL-40 correlated with adipsin and MMP-9, markers that were also found to be increased in subjects with asbestosis in the present study. Plasma adipsin has been shown to be associated with the degree of lung fibrosis in asbestos-exposed subjects [20]. The association of YKL-40 and adipsin has not been reported before. Our finding on the association of YKL-40 with MMP-9 is supported by findings in previous in vitro studies. YKL-40 has been shown to stimulate MMP-9 synthesis in BAL alveolar macrophages from smoking COPD patients [42] and in human fibroblasts from nasal mucosa [43]. Moreover, in cultured murine macrophages, YKL-40 has been reported to stimulate MMP-9 expression and inhibition of YKL-40 with siRNA was found to decrease MMP-9 expression [44].

In the present study, subjects with asbestosis had on average a mild disease with well-preserved lung function and had not yet developed severe restrictive lung function which can be regarded as a limitation of the study. Only *D*_L,CO_ was significantly lower in asbestosis subjects. However, at present, this is characteristic to asbestosis diagnosed in industrialized countries: most cases of asbestosis are detected in an early phase when they show up only on radiological examinations prior to possible progression to severe restriction or respiratory insufficiency [45]. The asbestos-exposed group without asbestosis may well develop asbestosis in the coming years, although time after the beginning of the exposure was similar in both groups. Our finding on the increased YKL-40 levels in subjects with asbestosis compared to subjects who had not developed lung fibrosis may thus well reflect the early fibrotic changes detected by HRCT. Furthermore, we present here correlations of YKL-40 with known inflammatory and fibrotic markers, which may reflect interesting possible mechanisms in the pathogenesis of asbestosis. Additional experimental studies are needed to confirm if the observed correlations are translated to causality. However, previous results in the scientific literature do suggest that YKL-40 could also play a role in the pathogenesis of fibrotic changes and the findings presented in our clinical cohort support these intriguing hypotheses.

Improved biomarkers and treatment options are needed in the diagnostics and management of fibrotic pulmonary diseases. According to our findings, YKL-40 could be a potential novel biomarker for fibrosis and inflammation in asbestosis.

## Figures and Tables

**Table 1:** Subject characteristics.

	Exposed					Unexposed	
	No fibrosis		Asbestosis			Healthy controls	
*N*	66		19			28	
Age (years)	64.5	(6.2)	68.5	(6.0)	*p* = 0.015	62.2	(6.6)
Time since the beginning of the exposure (years)	43.6	(0.9)	46.2	(3.4)	*p* = 0.464		
FVC (% pred)	88.8	(15.6)	83.7	(10.8)	*p* = 0.194	99.5	(10.8)
FEV_1_ (% pred)	88.5	(14.2)	81.8	(12.0)	*p* = 0.071	96.7	(11.6)
*D*_L,CO_ (% pred)	106.4	(17.4)	87.1	(16.8)	*p* < 0.001	N.A.	
IL-6 (pg/ml)	3.2	(2.4–4.3)	3.2	(2.0–5.0)	*p* = 0.728	2.1	(1.6–2.8)
CRP (*μ*g/ml)	0.93	(0.37–1.99	1.20	(0.60–2.94)	*p* = 0.282	0.47	(0.27–1.00)
Adipsin (ng/ml)	893	(777–1069)	1022	(950–1224)	*p* = 0.004	908	(757–1045)
MMP-9 (ng/ml)	28.1	(22.4–36.9)	54.7	(32.8–70.9)	*p* < 0.001	30.3	(25.4–38.9)

Values are presented as mean (SD) or median (IQR) when appropriate according to the distribution of variables. FVC: forced vital capacity; FEV_1_: forced expiratory volume in 1 second; *D*_L,CO_: diffusing capacity for carbon monoxide; IL-6: interleukin 6; CRP: C-reactive protein; MMP-9: matrix metalloproteinase-9; N.A: not assessed. *p* values were calculated between asbestos-exposed subjects without fibrosis and asbestos-exposed subjects with asbestosis using unpaired *t*-test or Mann–Whitney *U* test when appropriate.

**Table 2:** Correlations between YKL-40 and other parameters in asbestos-exposed subjects (*n* = 85).

	YKL-40	
Age (years)	*r* = 0.102	*p* = 0.355
FVC (% pred)	*r* = −0.259	*p* = 0.018
FEV_1_ (% pred)	*r* = −0.240	*p* = 0.028
*D*_L,CO_ (% pred)	*r* = 0.127	*p* = 0.246
IL-6 (pg/ml)	rho = 0.314	*p* = 0.003
CRP (*μ*g/ml)	rho = 0.371	*p* < 0.001
Adipsin (ng/ml)	rho = 0.459	*p* < 0.001
MMP-9 (ng/ml)	rho = 0.243	*p* = 0.025

Pearson's (*r*) or Spearman's (rho) correlation coefficient was used according to the distribution of variables. FVC: forced vital capacity; FEV_1_: forced expiratory volume in 1 second; *D*_L,CO_: diffusing capacity for carbon monoxide; IL-6: interleukin 6; CRP: C-reactive protein; MMP-9: matrix metalloproteinase-9.
